# Development of predictive equation and score for 5-year metabolic syndrome incidence in Japanese adults

**DOI:** 10.1371/journal.pone.0284139

**Published:** 2023-04-07

**Authors:** Anwar Ahmed Salim, Shin Kawasoe, Takuro Kubozono, Satoko Ojima, Takeko Kawabata, Hiroshi Hashiguchi, Yoshiyuki Ikeda, Masaaki Miyata, Hironori Miyahara, Koichi Tokushige, Yoshihiko Nishio, Mitsuru Ohishi

**Affiliations:** 1 Department of Cardiovascular Medicine and Hypertension, Graduate School of Medical and Dental Sciences, Kagoshima University, Kagoshima, Japan; 2 Department of Diabetes and Endocrine Medicine, Graduate School of Medical and Dental Sciences, Kagoshima University, Kagoshima, Japan; 3 School of Health Sciences, Faculty of Medicine, Kagoshima University, Kagoshima, Japan; 4 Kagoshima Kouseiren Hospital, Kagoshima, Japan; Endocrinology and Metabolism Population Sciences Institute, Tehran University of Medical Sciences, ISLAMIC REPUBLIC OF IRAN

## Abstract

**Background:**

Predicting metabolic syndrome (MetS) is important for identifying high-risk cardiovascular disease individuals and providing preventive interventions. We aimed to develop and validate an equation and a simple MetS score according to the Japanese MetS criteria.

**Methods:**

In total, 54,198 participants (age, 54.5±10.1 years; men, 46.0%), with baseline and 5-year follow-up data were randomly assigned to ‘Derivation’ and ‘Validation’ cohorts (ratio: 2:1). Multivariate logistic regression analysis was performed in derivation cohort and scores were assigned to factors corresponding to β-coefficients. We evaluated predictive ability of the scores using area under the curve (AUC), then applied them to validation cohort to assess reproducibility.

**Results:**

The primary model ranged 0–27 points had an AUC of 0.81 (sensitivity: 0.81, specificity: 0.81, cut-off score: 14), and consisted of age, sex, blood pressure (BP), body mass index (BMI), serum lipids, glucose measurements, tobacco smoking, and alcohol consumption. The simplified model (excluding blood tests) ranged 0–17 points with an AUC of 0.78 (sensitivity: 0.83, specificity: 0.77, cut-off score: 15) and included: age, sex, systolic BP, diastolic BP, BMI, tobacco smoking, and alcohol consumption. We classified individuals with a score <15 and ≥15 points as low- and high-risk MetS, respectively. Furthermore, the equation model generated an AUC of 0.85 (sensitivity: 0.86, specificity: 0.55). Analysis of the validation and derivation cohorts yielded similar results.

**Conclusion:**

We developed a primary score, an equation model, and a simple score. The simple score is convenient, well-validated with acceptable discrimination, and could be used for early detection of MetS in high-risk individuals.

## Introduction

Metabolic syndrome (MetS) develops from underlying risk factors mainly abdominal obesity and insulin resistance, accompanied by metabolic risk factors such as physical inactivity, genetic predisposition, increasing age, atherogenic dyslipidemia, elevated blood pressure and plasma glucose, and prothrombotic and proinflammatory states [1–3]. The primary reason to promote MetS public awareness is the rising global MetS prevalence that leads to increased risk of developing atherosclerotic cardiovascular diseases and increased cardiovascular disease (CVD) morbidity and mortality among MetS individuals [3, 4]. The global prevalence of MetS in adults is estimated to be around 20–25% [5]. In the United States from 2007 to 2014, the prevalence of MetS in adults was 34.3% [6] while in Japan, based on the National Integrated Project for Prospective Observation of Non-communicable Disease And its Trends in Aged (NIPPON DATA) (1990–2005), the age-adjusted prevalence of MetS was 19.3% [7].

MetS prevalence and CVD risk associated with MetS depend on ethnic variation and the MetS definition used in individual populations [8–10]. The Japanese criteria for MetS differ from other available guidelines, such as the World Health Organization (WHO), International Diabetes Federation (IDF), National Cholesterol Education Program Adult Treatment Panel-III (NCEP ATP-III), and American Heart Association/National Heart, Lung, and Blood Institute (AHA/NHLBI) [11, 12], by including waist circumference (WC) as an essential component, with unique WC cut-off points [13]. Even though the IDF had suggested ethnic-specific WC cut-off points of ≥ 90 cm for men and ≥ 80 cm for women in Asian populations, accumulated evidence obtained using computed tomography scans indicate a visceral fat area of 100 cm^2^ at the navel level is equivalent to a WC of ≥ 85 cm for men and ≥ 90 cm for women [14]. These are associated with increased MetS morbidity in Japan [15]. Furthermore, the WC concept was easily utilized by the public as part of the Japan national health screening and intervention campaign for MetS launched in 2008 [16]. Although risk prediction models are becoming more prevalent in healthcare practice due to their increasing importance in early disease detection, intervention, and decision-making [17–19], MetS risk prediction models are still inconsistent. A 3-year study conducted by Obokata and colleagues [20] identified nine independent variables that predicted the MetS recovery and incident MetS with c-statistics of 0.70 and 0.80 respectively. A study in China developed a 4-year prediction model containing five predictors of MetS with a c-statistic of 0.78 [21]. Although previous reports tried to develop MetS prognostic models for different populations [22–25], the majority lacked validation, were cross-sectional studies, had a smaller sample size, did not involve lifestyle factors, and none used Japanese criteria for MetS [26], except for the Japanese Metabolic Syndrome Risk Score questionnaire, which was developed as a screening tool for diagnosing MetS and insulin resistance in Japan.

Prognostic tools aid in identifying individuals with a high risk for MetS, thus, giving them an opportunity to improve their health. To achieve this goal, the use of screening parameters and cut-off values proposed for a specific population is essential. Therefore, the current study aimed to determine independent predictors of MetS, develop and validate an equation, and develop a simple prognostic model for 5-year MetS risk in the adult Japanese population using the Japanese MetS definition criteria.

## Materials and methods

### Source of data and participants

This retrospective cohort study analyzed the annual health check-up data of 198,292 individuals from Kagoshima Kouseirin Hospital from October 2008 to March 2019. We included participants aged 30–69 years, with baseline and follow-up data at Year 5 (range, 3–7 years). We excluded all participants with MetS at baseline and those with missing data required for statistical analysis. The remaining 54,198 participants were randomly assigned in a ratio of 2:1 to derivation cohort, used to develop a risk score for MetS and validation cohort, used to evaluate the validity of the score. The data were anonymized, and all participants were given the option to opt out of the study. This study complied with the Declaration of Helsinki and was approved by the institutional ethics committee of the Graduate School of Medical and Dental Sciences, Kagoshima University (IRB Approval number: 520) and Kagoshima Kouseiren Hospital (IRB Approval number: 168).

### Data collection and candidate predictors

During annual health checkups, a self-administered questionnaire was used to collect data on medications for hypertension, diabetes mellitus (DM), dyslipidemia, and lifestyle factors. Participants were divided into four age groups: 30–39 years, 40–49 years, 50–59 years, and 60–69 years. Tobacco smoking and alcohol consumption habits were grouped into tobacco smoking and non-tobacco smoking (those without a history of smoking or had smoked in the past); habitual alcohol consumption (those who consumed alcohol daily) and occasional alcohol consumption (those who consumed alcohol rarely or sometimes), respectively. An exercise habit was defined as having regular exercise regimen of at least 30 min per week. Anthropometric measurements were obtained using WHO standard operating procedures [27]. Body mass index (BMI) was calculated as body weight in kilograms divided by height squared in meters. BMI was divided into six groups: < 21.0 kg/m^2^, 21.0–22.9 kg/m^2^, 23.0–24.9 kg/m^2^, 25.0–26.9 kg/m^2^, 27.0–28.9 kg/m^2^, and ≥ 29.0 kg/m^2^. Brachial blood pressure (BP) was measured in a seated position after 3–5 min of rest in a quiet room with an appropriately sized cuff on the right arm while the elbow rested on a desk with the mid-arm at heart level. A well-trained staff member recorded BP measurements during enrollment and subsequent visits. Systolic BP (SBP) was divided into four groups: < 120 mmHg, 120–129 mmHg, 130–139 mmHg, and ≥ 140 mmHg; diastolic BP (DBP) was divided into 3 groups: < 80 mmHg, 80–89 mmHg, and ≥ 90 mmHg. The BP readings were recorded once during the annual health checkup. Blood samples were collected after an overnight fast. Serum triglyceride (TG), low-density lipoprotein cholesterol (LDL-C), high-density lipoprotein cholesterol (HDL-C), and fasting plasma glucose (FPG) levels were measured using the standard laboratory procedures. TG was divided into three groups: <100 mg/dL, 100–149 mg/dL, and ≥ 150 mg/dL; HDL-C into three groups: ≥ 60 mg/dL, 40–59 mg/dL, and < 40 mg/dL; LDL-C into three groups: < 100 mg/dL, 100–139 mg/dL, and ≥ 140 mg/dL; and FPG into two groups: <110 mg/dL and ≥ 110 mg/dL. DM was defined as an FPG ≥ 126 mg/dL or use of anti-diabetic medication. Dyslipidemia was defined as elevated serum LDL-C ≥ 140 mg/dL, elevated serum TG ≥ 150 mg/dL, decreased serum HDL-C < 40 mg/dL, or use of lipid-lowering medications.

### Outcome

MetS was defined based on the Japanese diagnostic criteria for MetS [28]. This criterion indicates that MetS is present if there is increased visceral fat accumulation expressed by WC of ≥ 85 cm for men and ≥ 90 cm for women, with at least two of the following risk factors: (1) dyslipidemia, elevated serum TG ≥ 150 mg/dL, low HDL-C < 40 mg/dL, or using antidyslipidemic medications; (2) elevated BP: SBP ≥ 130 mmHg, DBP ≥ 85 mmHg, or using antihypertensive medications; and (3) hyperglycemia, FPG ≥ 110 mg/dL, or on anti-diabetic medication. The outcome was MetS incidence after 5-years of follow-up.

### Statistical analysis

Continuous variables including age, BMI, SBP, DBP, LDL-C, and HDL-C were expressed as mean ± standard deviation (SD), except for TG and FPG, which were expressed as medians with interquartile range. Categorical variables, including sex, dyslipidemia, DM, tobacco smoking, alcohol consumption, and exercise habits were presented as numbers and percentages. We compared the characteristics between derivation and validation cohorts using the Chi-square test, Student’s unpaired t-test, and Wilcoxon rank-sum test for categorical, normally distributed continuous, and skewed-continuous distribution variables, respectively.

A logistic regression analysis was performed for each variable. Odds ratios (ORs) and 95% confidence intervals (CIs) were used to estimate the incidence of MetS. The primary model was adjusted to obtain significant risk factors associated with MetS and used to create a risk score for estimating the 5-year incidence of MetS. We assigned each category of risk factor corresponding to the β coefficients of multivariate logistic regression, in accordance with the methods used in previous investigations of risk score, including the Japan Epidemiology Collaboration on Occupational Health Study group: 1, β = 0.01–0.20; 2, β = 0.21–0.80; 3, β = 0.81–1.20; 4, β = 1.21–2.20; and 5, β > 2.20 [29, 30]. The reference category for each risk factor was assigned a score of 0. The total risk score for MetS incidence was calculated as the sum of risk factor score points.

To obtain a simple score with fewer commonly used risk components that can be easily applied in practical or screening settings, we decided to include candidate variables that do not require blood sampling. The simple score included the following seven risk candidates from the primary score: BMI, age, sex, SBP, DBP, tobacco smoking, and alcohol consumption. Statistical analysis methods like those used for the primary score were performed using derivation cohort to assess the association between risk factor candidates (not requiring blood) and MetS development. Furthermore, we developed an equation to predict the probability of MetS in 5 years using the candidate risk factors from the primary score; age, sex, BMI, SBP, DBP, TG, LDL-C, HDL-C, FPG, tobacco smoking, and alcohol consumption. All variables were treated as continuous except for sex, tobacco smoking, and alcohol consumption, which were treated as binary variables. The input for tobacco smoking and alcohol consumption were coded as 1 for a yes response and 0 for a no response. We applied all the scoring methods to the validation cohort and performed receiver operating characteristic (ROC) analysis to assess discrimination. A sensitivity analysis was performed using the bootstrap resample method. The 95% bootstrap confidence interval of the odds ratios in multivariable logistic regression analysis were calculated based on 2,500 bootstrap resamples. Finally, we developed the calibration plots to assess the agreement between the MetS predictions and observations for the equation model in the derivation and validation cohorts. All statistical analyses were performed using JMP Pro version 15 (SAS Institute, Inc., Cary, NC, USA), and calibration was performed with R version 4.2.2. (The R Foundation for Statistical Computing, Vienna, Austria) using Package ‘rms’. Statistical significance was set at P <0.05.

## Results

### Baseline characteristics

**Fig 1:**
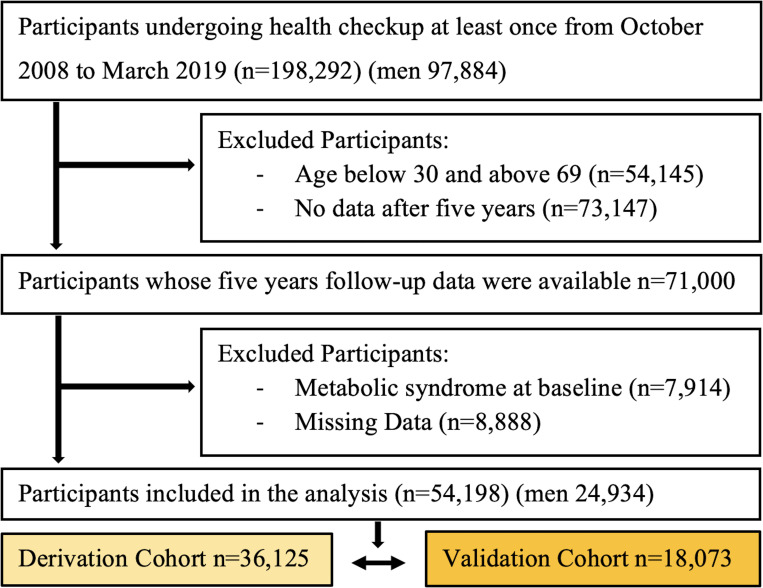
Study population. A total of 54,198 (men 24,934) selected participants classified into derivation cohort (n = 36,125) and validation cohort (n = 18,073).

This study included a total of 54,198 participants (mean [±SD] age, 54.5 ± 10.1 years; men, 46%), with a 5-year follow-up period, who were randomly divided into derivation (36,125 participants) and validation (18,073 participants) cohorts in a ratio of 2:1, based on inclusion and exclusion criteria, as shown in Fig 1. The baseline characteristics of the two cohorts are presented in Table 1. No statistically significant differences were observed between groups. A total of 2,326 (6.4%) and 1,216 (6.7%) patients in derivation and validation cohorts developed MetS, respectively.

**Table 1:** Baseline characteristics of the study population in derivation and validation cohorts.

	Derivation Cohort N = 36,125	Validation Cohort N = 18,073	P-value
Age, years	54.6 ± 10.1	54.5 ± 10.1	0.34
Men, n (%)	16616 (46.0)	8318 (46.0)	0.95
BMI, kg/m2	22.8 ± 3.1	22.9 ± 3.1	0.69
SBP, mmHg	122.9 ± 18.0	122.8 ± 17.9	0.59
DBP, mmHg	75.8 ± 10.9	75.7 ± 10.9	0.99
Diabetes, n (%)	1293 (3.6)	684 (3.8)	0.23
Dyslipidemia, n (%)	13456 (37.3)	6759 (37.4)	0.73
Tobacco Smoking, n (%)	7076 (19.6)	3483 (19.3)	0.38
Alcohol Consumption, n (%)	9466 (26.2)	4763 (26.4)	0.71
Exercise Habit, n (%)	10369 (28.7)	5130 (28.4)	0.44
Triglyceride, mg/dL	85 [62, 120]	86 [62, 120]	0.59
LDL-C, mg/dL	121.7 ± 30.5	121.8 ± 30.5	0.77
HDL-C, mg/dL	61.3 ± 14.8	61.4 ± 14.7	0.44
FPG, mg/dL	94 [88, 101]	94 [88, 101]	0.09

Baseline characteristics of the study population in derivation and validation cohorts. Continuous variables are expressed as mean ± standard deviation, except for triglycerides and FPG, which are expressed as median [1^st^ quartile, 3^rd^ quartile]. Categorical variables are expressed as numbers of subjects and proportions (percentage). Differences between derivation and validation cohorts for normally distributed continuous variables, skewed-distribution continuous variables, and categorical variables are analyzed using Student’s unpaired t-test, Wilcoxon test, and Chi-square test, respectively.

BMI, body mass index; SBP, systolic blood pressure; DBP, diastolic blood pressure; LDL-C, low-density lipoprotein cholesterol; HDL-C, high-density lipoprotein cholesterol; FPG, fasting plasma glucose.

### Association between risk factors and MetS

The risk factors associated with MetS are listed in Table 2. In the adjusted model, older age; being a man; higher BMI; elevated SBP and DBP; elevated TG, HDL-C, LDL-C, and FPG levels; tobacco smoking; and alcohol consumption were associated with increased risk of MetS. We initially adopted all the above-mentioned significant variables as candidate predictors. As the categories of candidate risk factors changed to higher values, the risk of developing MetS increased. BMI had the strongest risk compared to all other variables. A comparison with the reference group (BMI < 21.0 kg/m^2^), BMI 21.0–22.9 kg/m^2^ had the lowest risk (OR 4.07, P < 0.01), followed by BMI 23.0–24.9 kg/m^2^, BMI 25.0–26.9 kg/m^2^, and BMI 27.0–28.9 kg/m^2^, while BMI ≥ 29.0 kg/m^2^ had a marked increase in MetS risk (OR 45.25, P < 0.01). A higher number of cases with a 5-year MetS incidence were observed in men (10.4%) than in women (3%); men generally had increased risk of developing MetS (OR 3.0, P < 0.01) compared to women. Elevated serum TG was associated with the risk of MetS and serum TG level ≥ 150 mg/dL significantly increased MetS risk (OR 2.41, P < 0.01) compared to serum TG < 100 mg/dL. HDL-C < 40 mg/dL was associated with increased risk of MetS compared to both HDL-C 40–59 mg/dL and HDL-C ≥ 60 mg/dL. In addition, the risk of MetS significantly increased in participants with FPG ≥ 110 mg/dL (OR 2.47, P < 0.01). Categories of SBP were associated with an increased risk of MetS as SBP gradually increased. SBP ≥ 140 mmHg had the highest risk (OR 1.74, P < 0.01). All age categories > 40 years were significantly associated with MetS compared with individuals between 30–39 years old. Smoking and alcohol consumption were also associated with the development of MetS. In contrast, exercise habits were not associated with an increased MetS risk. The results of the sensitivity analysis using the bootstrap method are shown in supporting information (S1 Table). The odds ratios and 95% confidence intervals from the bootstrap method were similar to those from the Derivation cohort in the main analysis.

**Table 2:** Association between risk factor categories and 5-year metabolic syndrome incidence.

Risk factors		Number of subjects	Number of cases	Crude		Adjusted	
				OR (95% CI)	P-value	OR (95% CI)	P-value
Age, years	30–39	3712	199	Ref		Ref	
	40–49	7311	502	1.30 (1.10–1.54)	<0.01	1.32 (1.10–1.59)	<0.01
	50–59	10857	736	1.28 (1.09–1.51)	<0.01	1.41 (1.18–1.69)	<0.01
	60–69	14245	889	1.18 (1.00–1.38)	0.04	1.36 (1.13–1.64)	<0.01
Sex	Men	16616	1734	3.72 (3.38–4.10)	<0.01	2.66 (2.36–2.99)	<0.01
	Women	19509	592	Ref		Ref	
BMI, kg/m2	<21.0	10093	56	Ref		Ref	
	21.0–22.9	9810	314	5.93 (4.45–7.89)	<0.01	4.07 (3.05–5.43)	<0.01
	23.0–24.9	8309	626	14.60 (11.09–19.22)	<0.01	9.40 (7.11–12.43)	<0.01
	25.0–26.9	4523	630	29.00 (22.01–38.22)	<0.01	19.49 (14.71–25.82)	<0.01
	27.0–28.9	2017	365	39.06 (29.75–52.71)	<0.01	28.55 (21.30–38.28)	<0.01
	≥29.0	1373	335	57.84 (43.28–77.31)	<0.01	45.25 (33.51–61.10)	<0.01
SBP, mmHg	<120	15829	637	Ref		Ref	
	120–129	8047	691	2.24 (2.00–2.50)	<0.01	1.60 (1.41–1.82)	<0.01
	130–139	5915	410	1.78 (1.56–2.01)	<0.01	1.48 (1.27–1.73)	<0.01
	≥140	6334	588	2.44 (2.17–2.74)	<0.01	1.74 (1.48–2.05)	<0.01
DBP, mmHg	<80	22643	1052	Ref		Ref	
	80–89	9499	846	2.00 (1.83–2.0)	<0.01	1.32 (1.18–1.48)	<0.01
	≥90	3983	428	2.47 (2.20–2.78)	<0.01	1.34 (1.15–1.58)	<0.01
Triglycerides, mg/dL	<100	22401	779	Ref		Ref	
	100–149	8891	898	3.12 (2.82–3.44)	<0.01	1.82 (1.63–2.03)	<0.01
	≥150	4833	649	4.31 (3.00–3.60)	<0.01	2.41 (2.12–2.75)	<0.01
HDL-C, mg/dL	≥60	17981	668	Ref		Ref	
	40–59	16419	1389	2.0 (2.16–2.52)	<0.01	1.45 (1.23–1.70)	<0.01
	<40	1725	269	4.79 (4.12–5.57)	<0.01	1.59 (1.32–1.91)	<0.01
LDL, mg/dL	<100	8646	422	Ref		Ref	
	100–139	18056	1109	1.28 (1.14–1.43)	<0.01	1.05 (0.93–1.20)	0.37
	≥140	9423	795	1.80 (1.59–2.03)	<0.01	1.25 (1.09–1.44)	<0.01
FPG, mg/dL	<110	32812	1890	Ref		Ref	
	≥110	3313	436	2.48 (2.21–2.77)	<0.01	2.47 (2.18–2.79)	<0.01
Tobacco Smoking	No	29049	1599	Ref		Ref	
	Yes	7076	727	1.97 (1.79–2.15)	<0.01	1.39 (1.24–1.56)	<0.01
Alcohol Consumption	No	26659	1453	Ref		Ref	
	Yes	9466	873	1.76 (1.61–1.92)	<0.01	1.18 (1.06–1.31)	<0.01
Exercise Habit	Yes	10373	684	Ref		Ref	
	No	25752	1649	0.97 (0.89–1.07)	0.55	1.03 (0.93–1.14)	0.63

The OR and 95% CI of five-year MetS for each risk factor are calculated using logistic regression analysis. In the multivariate model, the OR is adjusted for the following variables: age, sex, BMI, DBP, SBP, triglycerides, LDL-C, HDL-C, FPG, tobacco smoking, alcohol consumption, and exercise habits.

BMI, body mass index; SBP, systolic blood pressure; DBP, diastolic blood pressure; LDL-C, low-density lipoprotein cholesterol; HDL-C, high-density lipoprotein cholesterol; FPG, fasting plasma glucose; OR odds ratio; CI, confidence interval; Ref, reference.

### Development of risk prediction score for MetS (Primary score)

The score points derived for each candidate predictor and overall risk score are presented in Table 3. The points assigned for each candidate predictor according to the β coefficients of multivariate logistic regression were as follows: the reference category was assigned the lowest score point of 0 for all candidate predictors. All categories of BMI ≥ 23.0 kg/m^2^ had the highest score (5 points), followed by men (3 points), TG (3 points), FPG (3 points), age (2 points), SBP and DBP (2 points each), HDL-C and LDL-C (2 points each), tobacco smoking (2 points), and alcohol consumption (1 point). From the risk calculation, the overall risk score ranged 0–27 points.

**Table 3:** Points assigned to risk factors in the primary risk score for predicting 5-year metabolic syndrome incidence.

Risk factors		β	Point
Age, years	30–39	Ref	0
	40–49	0.28	2
	50–59	0.34	2
	60–69	0.30	2
Sex	Women	Ref	0
	Men	0.98	3
BMI, kg/m^2^	<21	Ref	0
	21–22.9	1.40	4
	23–24.9	2.24	5
	25–26.9	2.97	5
	27–28.9	3.35	5
	≥29	3.81	5
SBP, mmHg	<120	Ref	0
	120–129	0.47	2
	130–139	0.39	2
	≥140	0.56	2
DBP, mmHg	<80	Ref	0
	80–89	0.28	2
	≥90	0.30	2
Triglycerides, mg/dL	<100	Ref	0
	100–149	0.60	2
	≥150	0.88	3
HDL-C, mg/dL	≥60	Ref	0
	40–59	0.12	1
	<40	0.50	2
LDL, mg/dL	<100	Ref	0
	100–139	0.06	1
	≥140	0.22	2
FPG, mg/dL	<110	Ref	0
	≥ 110	0.90	3
Tobacco Smoking	No	Ref	0
	Yes	0.33	2
Alcohol Consumption	No	Ref	0
	Yes	0.16	1

Risk factors are given points according to the β coefficients of the multivariate logistic regression as follows: 1, β = 0.01–0.20; 2, β = 0.21–0.80; 3, β = 0.81–1.20; 4, β = 1.21–2.20; and 5, β > 2.20. All the reference categories are assigned a score of 0.

BMI, body mass index; SBP, systolic blood pressure; DBP, diastolic blood pressure; LDL-C, low-density lipoprotein cholesterol; HDL-C, high-density lipoprotein cholesterol; FPG, fasting plasma glucose; OR odds ratio; CI, confidence interval; Ref, reference; β, standardized partial regression coefficient.

**Fig 2:**
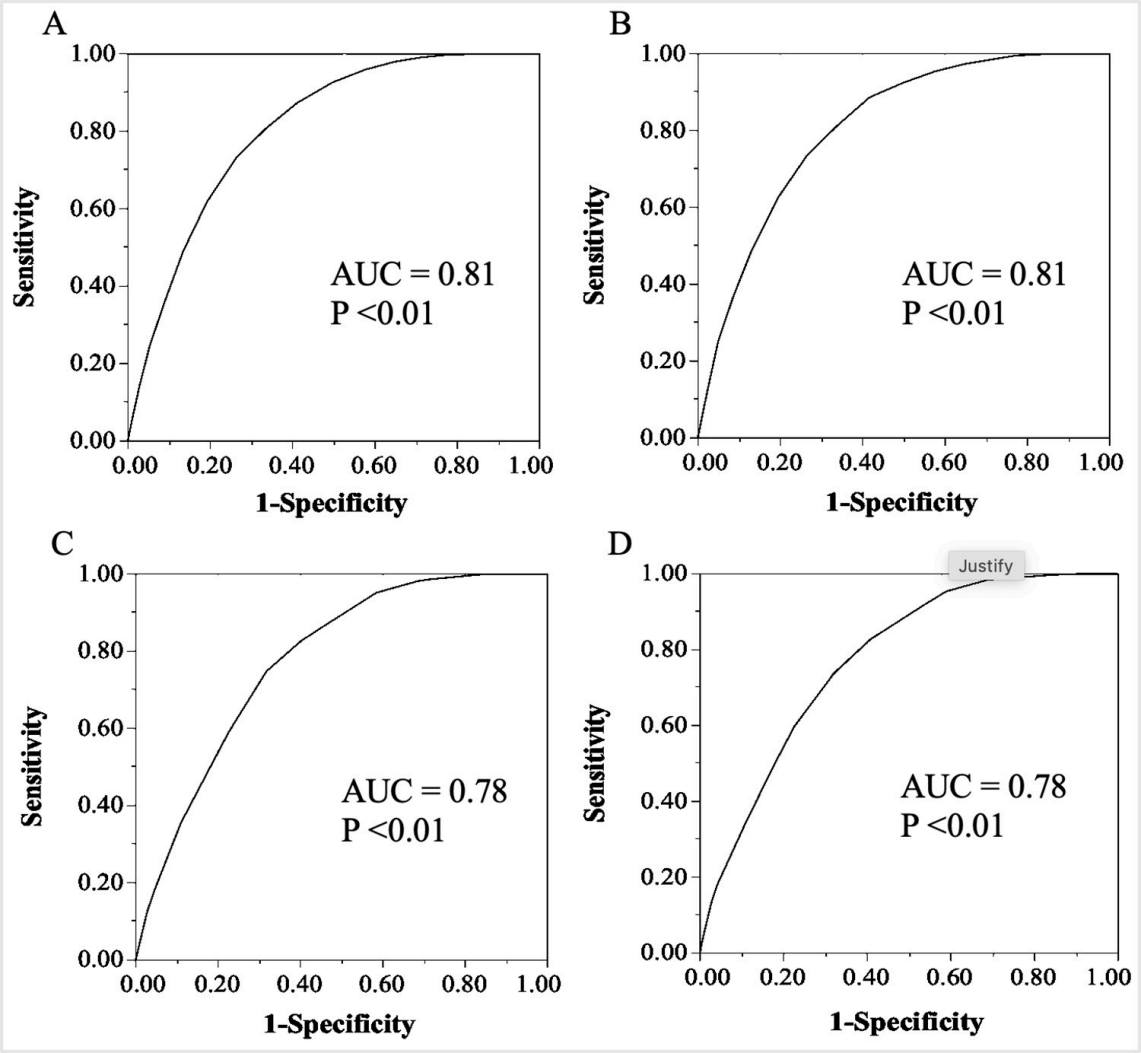
Five-year metabolic syndrome risk score prediction ability for the primary and the simple score model (excluding blood tests) in derivation and validation cohorts. The predictive ability of the primary and simple model was assessed using ROC curve analysis to determine the AUC for the derivation and validation cohorts. A. Primary score derivation cohort B. Primary score validation cohort C. Simple score derivation cohort, and D. Simple score validation cohort.

**Fig 3:**
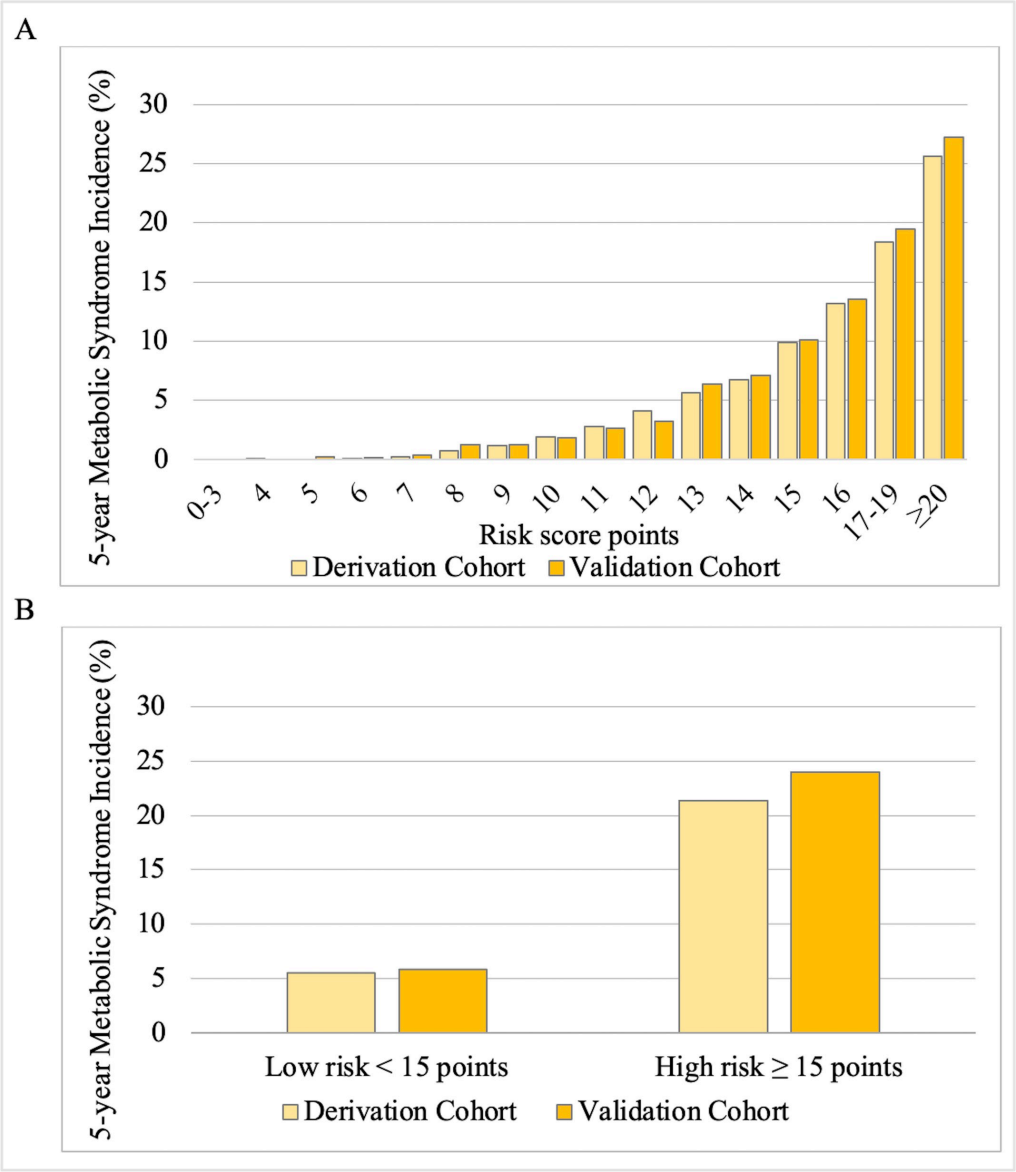
Proportions of metabolic syndrome in the primary and simple score model (excluding blood tests) in derivation and validation cohorts. Incidence of 5-year MetS according to the score in the derivation and validation cohort expressed in a bar graph. A. Primary score B. Simple risk score.

The ROC curve for predicting the incidence of MetS development generated an area under the curve (AUC) of 0.81 for derivation cohort as shown in Fig 2. The score predictive performance for the best cut-off point was calculated, as shown in Table 4. A score of 13 and 14 had the highest Youden index in derivation cohort with a sensitivity of 0.87 and specificity of 0.74 at score 13, and a sensitivity of 0.81 and specificity of 0.81 at score 14. The incidence of MetS development for each score in derivation cohort is shown in Fig 3. The risk gradually elevated (trend, P < 0.01) as the score increased. Up to a score of 7 points, the incidence of MetS remained below 1%. At a score of 15 points, the risk of developing MetS increased to 10%, and at an elevated score of ≥ 20 points, over 25% of the participants had developed MetS after 5-year follow-up. These results signify an incremental risk of MetS with an increasing total risk score.

**Table 4:** Predictive performance of the primary risk score for 5-year metabolic syndrome incidence in derivation and validation cohorts.

Derivation Cohort					
Risk score	Sensitivity	Specificity	PPV	NPV	Youden Index
5	0.99	0.17	0.03	1.32	0.17
6	0.99	0.23	0.04	1.35	0.23
7	0.99	0.29	0.07	1.26	0.29
8	0.99	0.35	0.07	1.22	0.35
9	0.99	0.42	0.08	1.20	0.41
10	0.98	0.50	0.10	1.18	0.48
11	0.96	0.59	0.13	1.16	0.55
12	0.93	0.66	0.16	1.11	0.59
13	0.87	0.74	0.17	1.09	0.61
14	0.81	0.81	0.20	1.07	0.61
15	0.73	0.87	0.25	1.04	0.60
16	0.62	0.91	0.29	1.01	0.53
17	0.49	0.95	0.32	0.99	0.44
18	0.36	0.97	0.38	0.97	0.33
19	0.25	0.98	0.44	0.96	0.23
20	0.15	0.99	0.49	0.95	0.14
Validation Cohort					
Risk score	Sensitivity	Specificity	PPV	NPV	Youden Index
5	1.00	0.17	0.03	1.32	0.17
6	1.00	0.23	0.04	1.35	0.23
7	1.00	0.29	0.07	1.26	0.29
8	0.99	0.35	0.07	1.20	0.34
9	0.98	0.42	0.08	1.20	0.41
10	0.97	0.50	0.10	1.18	0.47
11	0.95	0.58	0.13	1.15	0.54
12	0.92	0.66	0.15	1.12	0.58
13	0.89	0.74	0.17	1.09	0.62
14	0.81	0.81	0.20	1.07	0.62
15	0.73	0.87	0.25	1.04	0.61
16	0.63	0.91	0.31	1.00	0.54
17	0.49	0.95	0.31	0.99	0.44
18	0.37	0.97	0.39	0.97	0.34
19	0.26	0.99	0.44	0.95	0.24
20	0.15	0.99	0.48	0.94	0.14

PPV, positive predictive value; NPV, negative predictive value.

### Validation of risk prediction score

The ROC curve for validation cohort was similar to that for derivation cohort. The risk score had an AUC of 0.81 in validation cohort (Fig 2). The predictive performances of various cut-off points for validation cohort are shown in Table 4. Similar to derivation cohort, a score of 13 and 14 had the highest Youden index with a sensitivity of 0.89 and specificity of 0.74 at score 13, and a sensitivity of 0.81 and specificity of 0.81 at score 14. The incidence of MetS was comparable between the cohorts. In validation cohort similar to that in derivation cohort, as the score increased, the incidence of MetS also increased (trend, P < 0.01) (Fig 3).

### Consideration of a simple MetS score

The simple risk score included seven risk candidates: BMI, age, sex, SBP, DBP, alcohol consumption, and tobacco smoking. The scores ranged from 0–17 points (Table 5). The AUC was 0.78 (Fig 2) for derivation cohort and score predictive performance for the best cut-off point was calculated as shown in Table 6. A score of 15 had the highest Youden index in derivation cohort, with a sensitivity of 0.83 and specificity of 0.77. Similar findings were obtained in validation cohort for the simple score. Based on simple risk score, the 5-years incidence risk of MetS was below 10% for scores < 15 points and above 20% for scores ≥ 15 points, as presented in Fig 3. These results suggest a score of < 15 points as a 5-year low-risk of MetS and ≥ 15 points as a 5-year high-risk of MetS.

**Table 5:** Points assigned to risk factor in simple risk score (excluding blood tests) for predicting the 5-year metabolic syndrome incidence.

Risk factors		β	Point
Age, years	30–39	ref	0
	40–49	0.31	2
	50–59	0.46	2
	60–69	0.39	2
Sex	Women	ref	0
	Men	1.2	3
BMI, kg/m2	<21	ref	0
	21–22.9	1.62	4
	23–24.9	2.58	5
	25–26.9	3.40	5
	27–28.9	3.86	5
	≥29	4.28	5
SBP, mmHg	<120	ref	0
	120–129	0.36	2
	130–139	0.31	2
	≥140	0.49	2
DBP, mmHg	<80	ref	0
	80–89	0.21	2
	≥90	0.25	2
Tobacco Smoking	No	ref	0
	Yes	0.40	2
Alcohol Consumption	No	ref	0
	Yes	0.10	1

Risk factors are given points according to the β coefficients of the multivariate logistic regression as follows: 1, β = 0.0–0.20; 2, β = 0.21–0.80; 3, β = 0.81–1.20; 4, β = 1.21–2.20; and 5, β > 2.20. All the reference categories are assigned a score of 0.

BMI, body mass index; SBP, systolic blood pressure; DBP, diastolic blood pressure; OR odds ratio; CI, confidence interval; Ref, reference; β, standardized partial regression coefficient.

**Table 6:** Predictive performance of a simple risk score (excluding blood tests) for the 5-year risk of metabolic syndrome in derivation and validation cohorts.

Derivation Cohort					
Risk score	Sensitivity	Specificity	PPV	NPV	Youden Index
5	1.00	0.02	0.02	1.08	0.02
6	1.00	0.10	0.01	5.53	0.10
7	1.00	0.11	0.08	1.11	0.11
8	1.00	0.16	0.01	1.52	0.16
9	1.00	0.19	0.06	1.15	0.19
10	1.00	0.36	0.03	1.67	0.31
11	0.99	0.38	0.15	1.18	0.37
12	0.98	0.47	0.08	1.25	0.46
13	0.94	0.56	0.15	1.16	0.50
14	0.91	0.68	0.15	1.20	0.59
15	0.83	0.77	0.26	1.10	0.61
16	0.75	0.84	0.27	1.05	0.59
17	0.59	0.89	0.29	1.01	0.48
Validation Cohort					
Risk score	Sensitivity	Specificity	PPV	NPV	Youden Index
5	1.00	0.02	0.01	1.10	0.02
6	1.00	0.10	0.01	5.25	0.10
7	1.00	0.11	0.07	1.10	0.10
8	1.00	0.16	0.01	1.51	0.16
9	1.00	0.19	0.06	1.15	0.19
10	1.00	0.31	0.03	1.67	0.31
11	0.99	0.37	0.14	1.18	0.36
12	0.98	0.47	0.08	1.25	0.45
13	0.94	0.56	0.14	1.17	0.50
14	0.91	0.68	0.16	1.20	0.59
15	0.82	0.77	0.26	1.11	0.60
16	0.74	0.84	0.28	1.05	0.58
17	0.60	0.89	0.29	1.01	0.49

PPV, positive predictive value; NPV, negative predictive value

### The equation for predicting MetS incidence in 5 years

**Fig 4:**
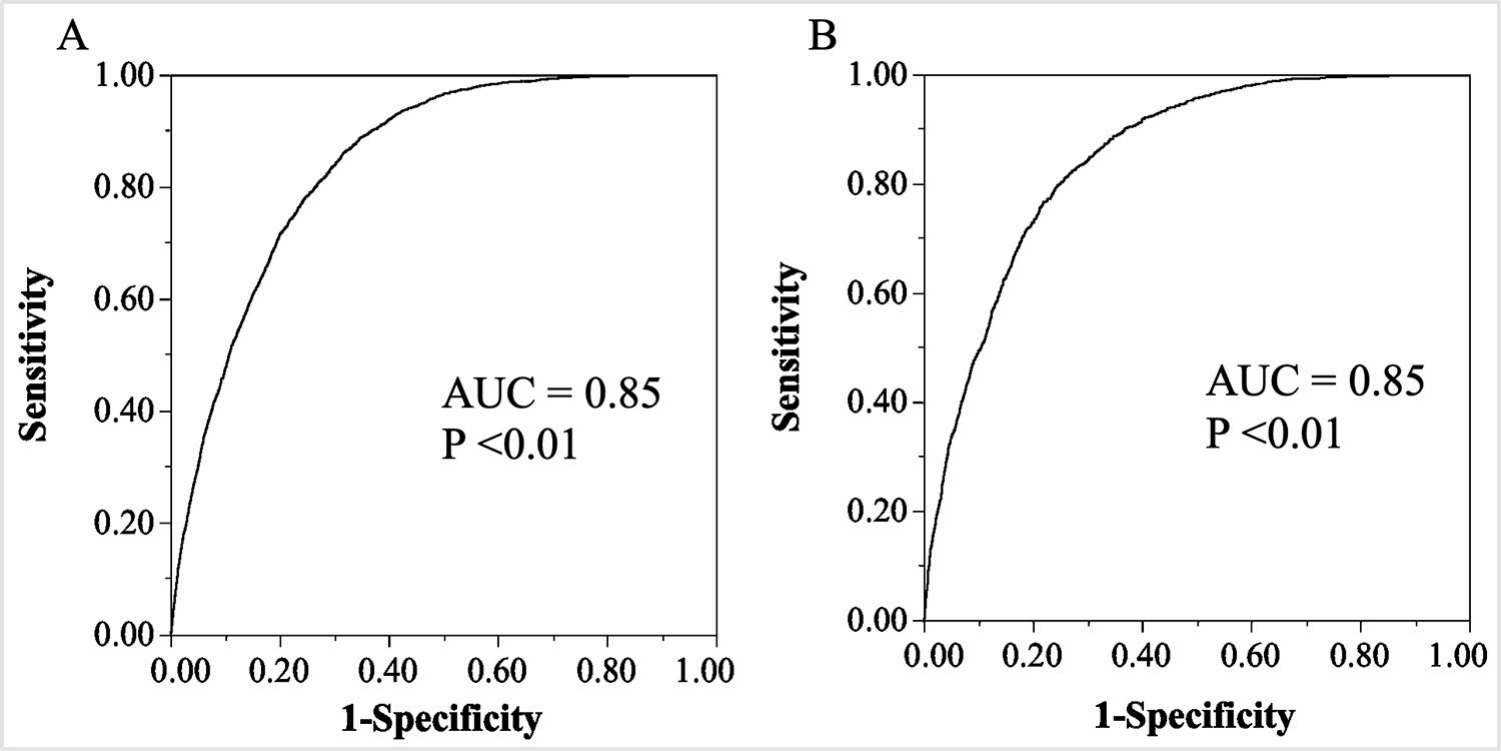
Five-year metabolic syndrome risk score prediction ability for the equation model in derivation and validation cohorts. The predictive ability of the equation model was assessed using ROC curve analysis to determine the AUC for the derivation and validation cohorts. A. Derivation cohort B. Validation cohort. AUC, area under the curve; ROC, receiver operating characteristic.

**Fig 5:**
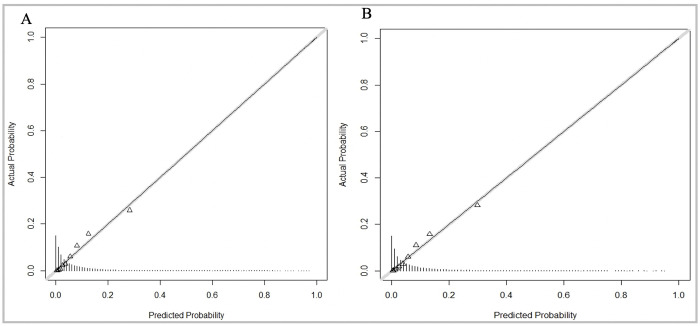
Calibration plots for the equation model in derivation and validation cohorts. The visual agreement between the MetS predictions (Predicted probability) and observations (Actual probability) for the equation model in the derivation and validation cohorts. A. Derivation cohort B. Validation cohort.

The equation to predict MetS incidence in 5 years from derivation cohort generated an AUC of 0.85 with a sensitivity of 0.86 and a specificity of 0.55. When applied to validation cohort, the AUC was 0.85 with a sensitivity of 0.87 and specificity of 0.56, similar to that of the derivation cohort as shown in Fig 4. The equation was well calibrated as shown in the calibration plot in Fig 5.

5-year MetS incidence Equation.

P = 1 / (1+ Exp (- (14.7123) + 0.0111 × age + 0.014 × FPG + 0.3135 × BMI + 0.0167 × DBP +

-0.0152 × HDL + 0.0040 ×LDL + 0.0083 × SBP + 0.0015 × TG + 0.2479 × alcohol consumption + 1.1735 × sex + 0.2049 × tobacco smoking)))

## Discussion

The present study created and validated an equation and a simple prognostic model for the 5-year risk of MetS in the general Japanese population. From the primary model that included blood sampling variables, we found 11 independent predictors of MetS, consisting of age, sex, SBP, DBP, BMI, TG, LDL-C, HDL-C, FPG, tobacco smoking, and alcohol consumption. The primary model was useful (AUC, 0.81) for predicting MetS. Similarly, the simplified model with excluded blood variables showed acceptable discrimination (AUC, 0.78) in predicting MetS. The continuous equation model had excellent predictive ability (AUC, 0.85) for MetS. In addition, the incidence of MetS gradually and simultaneously increased as the score increased in both derivation and validation cohorts. We recommend the application of simplified prediction score in clinical settings because it is easier to use with similar predictive ability as the primary model and has only seven variables that do not require the results of blood sampling. The equation model can be used for the near future generation of MetS screening software applications. To our knowledge, this is the first study to create an equation and a simplified prognostic score based on the Japanese MetS definition criteria to predict the 5-year risk of MetS.

MetS is associated with cardiovascular morbidity and mortality in Japan [31, 32] and globally [33]. Efforts to prevent CVD are dependent on the ability to evaluate and determine individual risk. There are few existing MetS prognostic models developed in other populations that use different MetS definition criteria for MetS outcome, including WHO, IDF, NCEP ATP-III, and AHA/NHLBI [8, 11, 12]; however, none of the previous studies have used the Japanese criteria for MetS. The WC cut-off points currently used in Japan were determined using the visceral fat area of 100 cm^2^ measured by computed tomography scans as a borderline for increased MetS morbidity, and it corresponds to a WC cut-off point of ≥ 85 cm in men and ≥ 90 cm in women, which is an essential part of the Japanese MetS definition [34].

After risk stratification, we found that age, sex, SBP, DBP, BMI, TG, LDL-C, HDL-C, FPG, tobacco smoking, and alcohol consumption were the common independent predictors of MetS. Furthermore, BMI emerged as the strongest predictor, followed by sex, FPG and serum TG levels. The difference in independent predictors of MetS across the various previous studies might be due to the differences in race, study design, and participants, although the risk factors identified in this study were generally consistent with those in the previous study [35, 36]. In this population, a slight increase in body weight led to a significant increase in MetS risk. The categories with BMI from 23.0–24.9 kg/m^2^ to 27.0–28.9 kg/m^2^ had a lower risk than BMI ≥ 29.0 kg/m^2^ after multivariate regression analysis, but both had similar scores of 5 points in the scoring system. An increase in BMI has been associated with an increase in WC, insulin resistance, and mortality [37, 38]; thus, this finding also underscores the importance of a lower BMI to attain lower WC cut-off points in this population. WC is an important indicator of visceral fat accumulation, which should be considered when predicting MetS. BMI and WC were strongly correlated (r = 0.85), and the existence of multicollinearity problems precluded both BMI and WC as factors in multivariate analyses. In this study, we decided to use BMI to predict MetS since WC has the disadvantage that it cannot be measured by oneself and that measurements fluctuate depending on body position, respiration, and measurement site.

A cohort study that created a score to predict the incidence and recovery from MetS used the harmonized definition of MetS to define MetS, wherein, WC criteria for Asian men (≥90 cm) and Asian women (≥80cm) was used^20)^. Interestingly, women had an increased risk of incident MetS in their study. In contrast, our study showed that men had an increased risk of incident MetS. On the other hand, our findings on sex were similar to a study that assessed lifestyle factors associated with MetS, in which Japanese MetS criteria were adapted [39]. Considering these results, different WC cut-off points, sample sizes, and different variables used in the multivariate regression may have influenced the sex risk for MetS results.

LDL-C, TG, and HDL-C were all found to increase the risk of MetS in the current study, however, elevated TG levels were found to be associated with higher risk of MetS than HDL-C and LDL-C levels. A previous study suggested similar findings that after a 2-year follow-up, TG had the highest AUC, indicating its significance in evaluating the risk of future MetS [40]. Additionally, other studies using decision tree analysis for predicting MetS also found TG to be an important feature for classifying MetS [41, 42]. Although atherogenic dyslipidemia is identified clinically by serum elevated LDL-C and TG levels and low HDL-C levels, hypertriglyceridemia has been independently associated with MetS and CVD. During the development of simple prognostic score, we did not include TG, but it is relevant to modify the TG levels using lifestyle modifications and medical therapy to reduce CVD risk [43, 44]. Elevated SBP was associated with increased risk of MetS relative to elevated DBP in our study, which is similar to previous studies [40] and different from another population study in China wherein DBP was associated with increased MetS risk relative to SBP [22]. Tobacco smoking and alcohol consumption was positively related to MetS similar to previous studies in Japan [45]. It is important to discourage young people from becoming tobacco smokers and to encourage tobacco cessation among smokers as risk of MetS could persist for 10 years or more among ex-smokers [46]. Our study showed no statistical association between exercise and 5-year MetS risk, similar to previous studies in Iran [47] and Japan [39]. This finding is because most individuals who have routine exercise habits are more likely of older age or have a preexisting health condition; thus, having no association between exercise habits and incidence MetS does not rule out the importance of exercise.

It is important to develop a risk score that is suitable and specific to a population. The present study used the Japanese MetS definition criteria to develop and validate a prognostic model for 5-year MetS risk in the general Japanese population. Although the developed primary model and equation model had a better AUC, we recommend the simple score model considering its convenience, low cost, and easy adaptation by healthcare practitioners. Based on the simple risk score, we suggest considering a score of <15 points as low-risk MetS and ≥15 points as high-risk MetS for classification. A threshold of 15 points or higher provides the most accurate prediction, and the percentage of individuals at risk for MetS markedly increases, as shown in Fig 3. Therefore, an intensive intervention targeting a group above 15 points may efficiently reduce future MetS with little effort and expense. In addition, this score can be used to emphasize lifestyle changes as key to reducing the risk of CVD and DM development. Lifestyle modifications, such as weight reduction, healthy diet, tobacco cessation, and increased physical activity may be initiated earlier after screening. We hope that our findings can assist clinicians in the early detection of MetS in high-risk individuals.

This study had several limitations. First, we only included participants who were involved in health checkups at a single facility in Japan; this may not be extrapolated to other ethnic groups. Second, the data were not collected prospectively, limiting our statistical analysis because most participants missed their annual health checkup visits. Therefore, the findings should be verified in further prospective observational studies. Third, we did not obtain BP readings on several occasions to assess sustained elevated BP, and future studies should monitor elevated BP.

## Conclusion

A 5-year simple MetS prognostic score model was developed and validated in the Japanese general population using the Japanese MetS criteria. The simple MetS score model includes age, sex, SBP, DBP, BMI, tobacco smoking, and alcohol consumption for examination, with acceptable discrimination. This model can be used clinically for screening high-risk individuals for MetS without requiring blood tests. The equation model had a good discrimination and calibration and can be integrated in MetS screening.

## Supporting information

S1 Table Mean odds ratio and 95% confidence intervals of multivariable logistic regression analysis from bootstrap resample method. The OR and 95% CI of five-year MetS for each risk factor calculated using the bootstrap sampling method to assess the points assigned to each factor. BMI, body mass index; SBP, systolic blood pressure; DBP, diastolic blood pressure; LDL-C, low-density lipoprotein cholesterol; HDL-C, high-density lipoprotein cholesterol; FPG, fasting plasma glucose; OR odds ratio; CI, confidence interval; Ref, reference.
(DOCX)
