# Supplementary material for: Development of predictive equation and score for 5-year metabolic syndrome incidence in Japanese adults
Source: PLoS One. 2023 Apr 7;18(4):e0284139. doi: 10.1371/journal.pone.0284139 (PMC10081753; doi:10.1371/journal.pone.0284139)
Supplement: S1 Table — The OR and 95% CI of five-year MetS for each risk factor calculated using the bootstrap sampling method to assess the points assigned to each factor. BMI, body mass index; SBP, systolic blood pressure; DBP, diastolic blood pressure; LDL-C, low-density lipoprotein cholesterol; HDL-C, high-density lipoprotein cholesterol; FPG, fasting plasma glucose; OR odds ratio; CI, confidence interval; Ref, reference. (DOCX) [file pone.0284139.s001.docx]

S1 Table. Mean Odds Ratio and 95% Confidence Intervals of Multivariable Logistic Regression analysis from Bootstrap Resample Method

| Risk factors | | OR (95% CI) |
| --- | --- | --- |
| Age, years | 30–39 | Ref |
|  | 40–49 | 1.32  (1.18–1.50) |
|  | 50–59 | 1.41  (1.25–1.59) |
|  | 60–69 | 1.35  (1.19–1.53) |
|  |  |  |
| Sex | Women | Ref |
|  | Men | 2.80  (2.59–3.3) |
| BMI, kg/m^2^ | <21 | Ref |
|  | 21–22.9 | 3.85  (3.18–4.78) |
|  | 23–24.9 | 9.28  (7.69–11.36) |
|  | 25–26.9 | 19.62  (16.26–23.97) |
|  | 27–28.9 | 29.26  (23.97–36.18) |
|  | $\geq$29 | 41.36  (33.60–51.14) |
|  |  |  |
| SBP, mmHg | <120 | Ref |
|  | 120–129 | 1.49  (1.36–1.61) |
|  | 130–139 | 1.40  (1.26–1.57) |
|  | ≥140 | 1.69  (1.50–1.89) |
|  |  |  |
| DBP, mmHg | <80 | Ref |
|  | 80–89 | 1.31  (1.21–1.41) |
|  | ≥90 | 1.36  (1.21–1.52) |
|  |  |  |
| Triglycerides, mg/dL | <100 | Ref |
|  | 100–149 | 1.83  (1.69–1.97) |
|  | ≥150 | 2.33  (2.12–2.54) |
|  |  |  |
| HDL-C, mg/dL | ≥60 | Ref |
|  | 40–59 | 1.18  (1.10–1.27) |
|  | <40 | 1.59  (1.40–1.81) |
|  |  |  |
| LDL, mg/dL | <100 | Ref |
|  | 100–139 | 0.99  (0.91–1.08) |
|  | ≥140 | 1.18  (1.08–1.30) |
|  |  |  |
| FPG, mg/dL | <110 | Ref |
|  | ≥ 110 | 2.53  (2.33–2.76) |
|  |  |  |
| Tobacco Smoking | No | Ref |
|  | Yes | 1.34  (1.24–1.45) |
|  |  |  |
| Alcohol Consumption | No | Ref |
|  | Yes | 1.18  (1.10–1.27) |
|  |  |  |
| Exercise Habit | Yes | Ref |
|  | No | 1.03  (0.93–1.14) |

The OR and 95% CI of five-year MetS for each risk factor calculated using the bootstrap sampling method to assess the points assigned to each factor.

BMI, body mass index; SBP, systolic blood pressure; DBP, diastolic blood pressure; LDL-C, low-density lipoprotein cholesterol; HDL-C, high-density lipoprotein cholesterol; FPG, fasting plasma glucose; OR odds ratio; CI, confidence interval; Ref, reference.
